# Facebook as a Pro-Ana and Pro-Mia resource

**DOI:** 10.1192/j.eurpsy.2021.1863

**Published:** 2021-08-13

**Authors:** G. Lladó Jordan, M.D.C. Díaz García, B. Lozano Díez, P. Mediavilla Sánchez, J.A. Gómez Del Barrio, R. Ayesa-Arriola

**Affiliations:** Idival, Valdecilla Biomedical Research Institute, Santander, Spain

**Keywords:** Facebook, Pro-Ana, eating disorders, Pro-Mia

## Abstract

**Introduction:**

INTRODUCTION Facebook is the world’s leading social network with 2,449 million users. Around 22 million of those users are registered in Spain, and 30% of them are aged between 16 and 31. Pro-Ana and Pro-Mia pages have found a space to promote Eating Disorders (ED) as a ‘lifestyle’ using their own code.

**Objectives:**

OBJECTIVE To study the characteristics of Pro-Ana and Pro-Mia Facebook profiles in Spanish.

**Methods:**

METHODS A non-computerized research of Facebook pages related to ED advocacy was conducted. The opened time, publications, photos, type of profiles (public/private) and link to a WhatsApp group of 58 Facebook pages were analyzed. A qualitative and descriptive analysis was carried out.

**Results:**

RESULTS From Facebook profiles: 62.07% contained ‘Ana’ in their profile name; 18.97% had been opened for more than 3 years; 79.31% had been shared; 48.28% mentioned Whatsapp groups; 91.38% were public profiles; 50% named other social networks; 75.86% added text to their publications; 25.86% had shared more than 20 photos on their profiles.

**Conclusions:**

CONCLUSIONS On platforms like Facebook, people with ED can: advocate for their disease, set up networks, share tips/tricks and encourage other users to become part of their community. Technological developments have made it easier to access to this type of resources. Despite the platform’s policy, there are still these kind of profiles that make a case for ED.

**Disclosure:**

No significant relationships.

